# Impacts of parent material on distributions of potentially toxic elements in soils from Pearl River Delta in South China

**DOI:** 10.1038/s41598-020-74490-2

**Published:** 2020-10-15

**Authors:** Qingye Hou, Zhongfang Yang, Tao Yu, Yuanhang You, Lei Dou, Kuo Li

**Affiliations:** 1 School of Earth Sciences and Resources, China University of Geosciences, Beijing, 100083 China; 2 3rd Geological Team, Guangdong Geological Bureau, Shaoguan, 512030 China; 3 Guangdong Geological Bureau, Guangdong Geologic Survey Institute, Guangzhou, 510080 China; 4 Institute of Geophysical and Geochemical Exploration, Chinese Academy of Geological Sciences, Langfang, 065000 China

**Keywords:** Geochemistry, Geochemistry

## Abstract

Assessing the impacts of parent material on distributions of potentially toxic elements (PTEs) in soils has significant consequences in the apportionment of their sources. In this study, geochemical distributions and sources of PTEs in the soils developed in quaternary sediments and granite plutons of Pearl River Delta (PRD), South China, were investigated. The results indicate that there are systematic differences between the concentrations of oxides and PTEs in the soils developed in these two parent materials. The parent material predominantly determines the element distributions in the soils. The PTEs of the deep soils developed in quaternary sediments originated mainly from mafic, felsic, and carbonate sources materials as well as polymetallic deposits. For the deep soils developed in granite plutons, the element associations are governed mainly by their geochemical affinities and behaviors and the mineral compositions of granite plutons. Anthropogenic activities impact the features of the PTEs in the surface soils of PRD. However, superimposed regional-scale pollution was found to not hide the effect of the parent material on the distribution of PTEs in the surface soils.

## Introduction

Geochemical distributions, sources, and environmental effects of potentially toxic elements (PTEs) in soils have gained significant attention over several years^1–5^. The soils in alluvial or flood plain have been intensively used for agriculture because of their generally high fertility^6^, and the alluvial plains have also undergone industrialization and urbanization for a long time in view of their topography and transportation condition. Therefore, the sources and risk assessment of PTEs in soils of alluvial plain have become hot topics of recent studies^7–11^. A river delta forms when a river carrying suspended sediments reaches an ocean, from which an alluvial plain forms. The PTEs in fluvial sediments originate from various sources in the catchment area and are transported by the river water and sediments. Alluvial soils developed in quaternary fluvial sediments could inherit the original geochemical characteristics of fluvial sediments. The geochemical distributions of PTEs in alluvial soils depend on the regional lithology of drainage basin, historical floodplain topography, and anthropogenic activities.

Pearl River Delta (PRD) in South China has undergone rapid industrialization and urbanization and become a major manufacturing hub over the past three decades. The sources and environmental impact of PTEs in surface soils, sediments, and groundwater of PRD have been of concern in recent years^12–17^. Anthropogenic activities have been identified as the most significant sources of PTEs in the surface soils of PRD, such as electronics and electroplating industries^12^, agricultural non-point sources^15^, atmospheric deposition^15^, and so on. However, the results of the regional geochemical survey indicated that the geochemical distributions of PTEs in the soils of PRD seemed to be associated with underlying parent material instead of man-made pollution^17^. The surface and deep soils in the alluvial plain of Pearl River contain high concentrations of soil organic carbon (SOC), TFe_2_O_3_ (total Fe, as Fe_2_O_3_), Mn, As, Cd, Co, Cr, Cu, Ni, Hg, Pb, and Zn^17^. For example, the median values of Mn, Cd, Cu and Hg concentrations in surface soils in the alluvial plain of Pearl River are 483 mg kg^−1^, 228 μg kg^−1^, 33.78 mg kg^−1^ and 136 μg kg^−1^ respectively. However, the median values of them in surface soils developed in granite plutons are 247 mg kg^−1^, 65 μg kg^−1^, 6.95 mg kg^−1^ and 77 μg kg^−1^ respectively. Therefore, the sources of the high concentrations of PTEs in the soils of PRD still remains unclear.

The total concentrations of PTEs are not enough to assess any actually occurring adverse effects on the soil ecosystem. The mobility and bioavailability of PTEs in the soils depend on their total concentrations, specific chemical form, binding state, metal properties, environmental factors, and soil properties^18^. Nevertheless, to our knowledge, whether there are differences in the mobility of PTEs in the soils developed in different parent materials has not been reported to date. Therefore, the assessment of the impacts of the parent material on the characteristics of PTEs in soils from an urbanization area deserves significant attention. It is also very useful to reevaluate the impact of human activities on the distributions of PTEs in the soils in these areas. In this study, we compared the geochemical characteristics of PTEs and the geochemical fractions of Cd and Pb in the soils developed in granite plutons and quaternary sediments in PRD. Our objectives were to (1) present the geochemical differences in the PTEs in surface soils (0–20 cm), deep soils (180–200 cm), and the soil vertical profiles developed in quaternary sediments and granite plutons in PRD, (2) discuss the factors affecting the distributions of PTEs, and (3) assess the major sources of PTEs in the soils from PRD.

## Results

### Element concentrations in surface and deep soils

The statistical summaries of each parameter as well as their skewness and kurtosis coefficients in the surface and deep soils can be found as Supplementary Tables S1, S2, S3, and S4 online.

**Figure 1:**
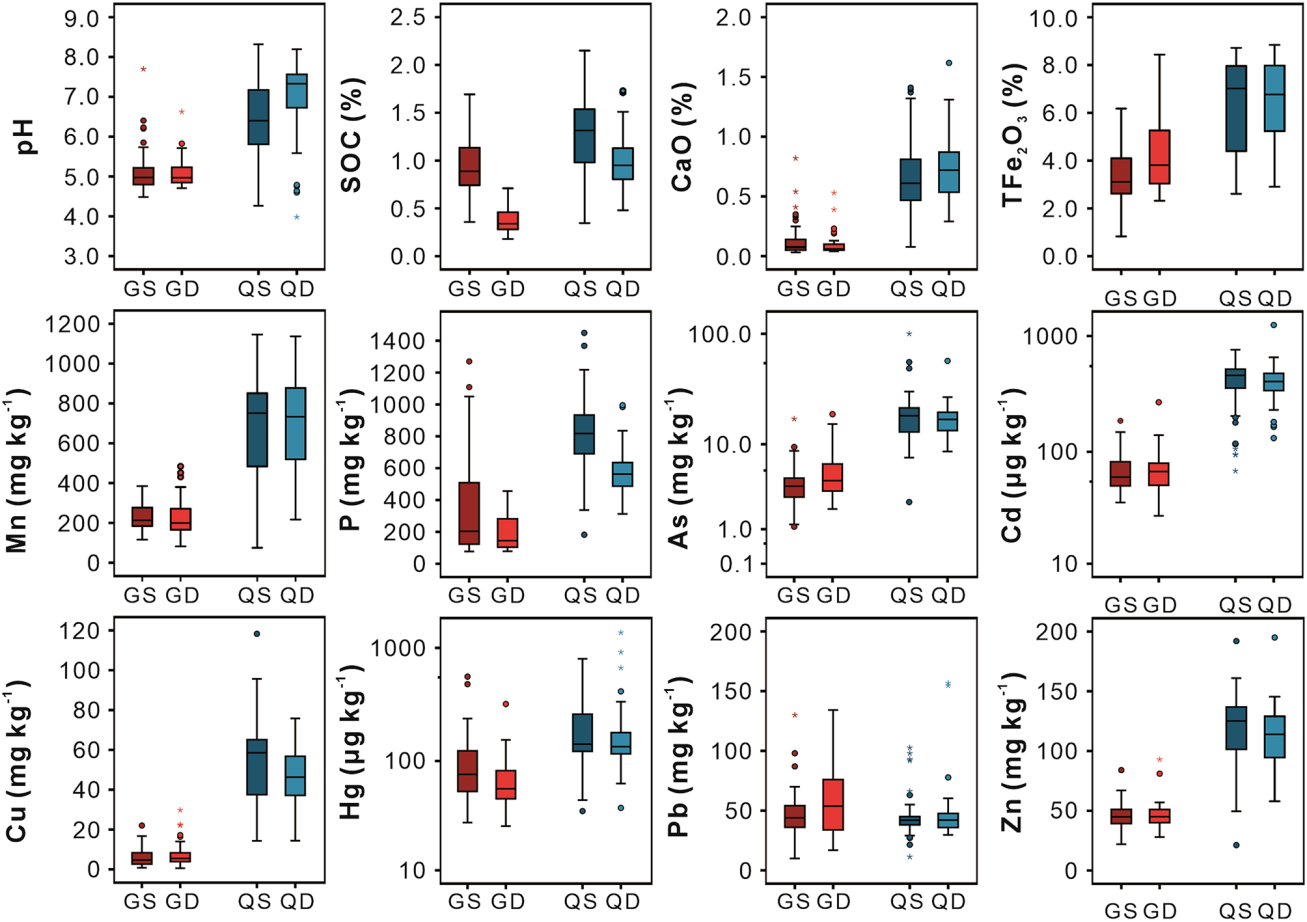
The Tukey boxplots of oxides and potential toxic elements of the surface and deep soils. QS and QD, the surface and deep soils developed in quaternary sediments; GS and GD, the surface and deep soils developed in granite plutons.

There are systematic differences in the concentrations of oxides and PTEs between the soils developed in the two parent materials. The concentrations of SOC, oxides, and PTEs of the surface and deep soils developed in the quaternary sediments are significantly higher than those of the soils developed in granite plutons, except for Pb (Fig. 1). For example, the median values of the concentration of Cd in the deep soils developed in the quaternary sediments and in granite plutons are 405 μg kg^−1^ (see Supplementary Table S2 online) and 67 μg kg^−1^ (see Supplementary Table S4 online), respectively.

**Figure 2:**
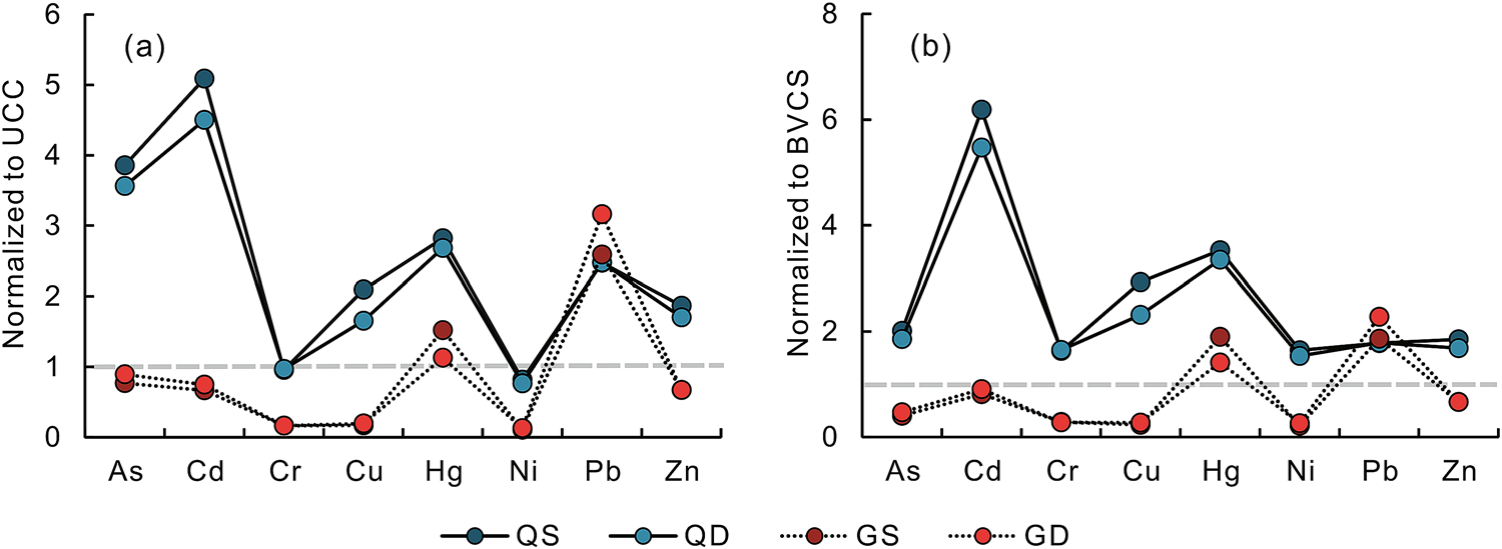
Normalized patterns for the potential toxic element contents in the soils. (**a**), upper continental crust (UCC) normalized data are from^19^; (**b**), background values of China soils (BVCS) normalized data are from^20^; QS and QD, the surface and deep soils developed in quaternary sediments; GS and GD, the surface and deep soils developed in granite plutons.

From a comparison with the composition of the upper continental crust (UCC)^19^ (see Supplementary Table S5 online), it is found that the concentrations of As, Cd, Cu, Hg, Pb, and Zn in the soils developed in the quaternary sediments are higher than the UCC abundances, and the concentrations of Cr and Ni are slightly lower than the UCC abundances (Fig. 2a). In the soils developed in granite plutons, the median values of the concentrations of As, Cd, Cr, Cu, Ni and Zn are lower than the UCC abundances (Fig. 2a). Normalized to the background values of China soils (BVCS)^20^ (Fig. 2b), all the PTEs in the soils developed in the quaternary sediments exhibit enriched features. Only the concentrations of Hg and Pb in the soils developed in granite plutons are slightly higher than the BVCS. Compared with the risk-screening values for soil contamination of agricultural land^21^, the concentrations of Cd and As in the deep soils developed in the quaternary sediments exceed the risk-screening values by 11.4% and 7.6%, respectively. The concentrations of Cd, As, Hg, and Pb in the surface soils developed in the quaternary sediments exceed the risk-screening values, and their rates reach 38.9%, 7.8%, 8.9%, and 1.1% respectively. All the PTEs in the soils developed in granite plutons do not exceed the risk-screening values. Further, the median values of the concentrations of PTEs in the deep soils developed in the quaternary sediments of PRD (see Supplementary Table S2 online) are significantly higher than those in the deep soils of other alluvial plains in China such as in the Yangtze^22^, Yellow^23^, and Haihe River Deltas^24^ (see Supplementary Table S5 online).

### PTEs in soil vertical profiles

**Figure 3:**
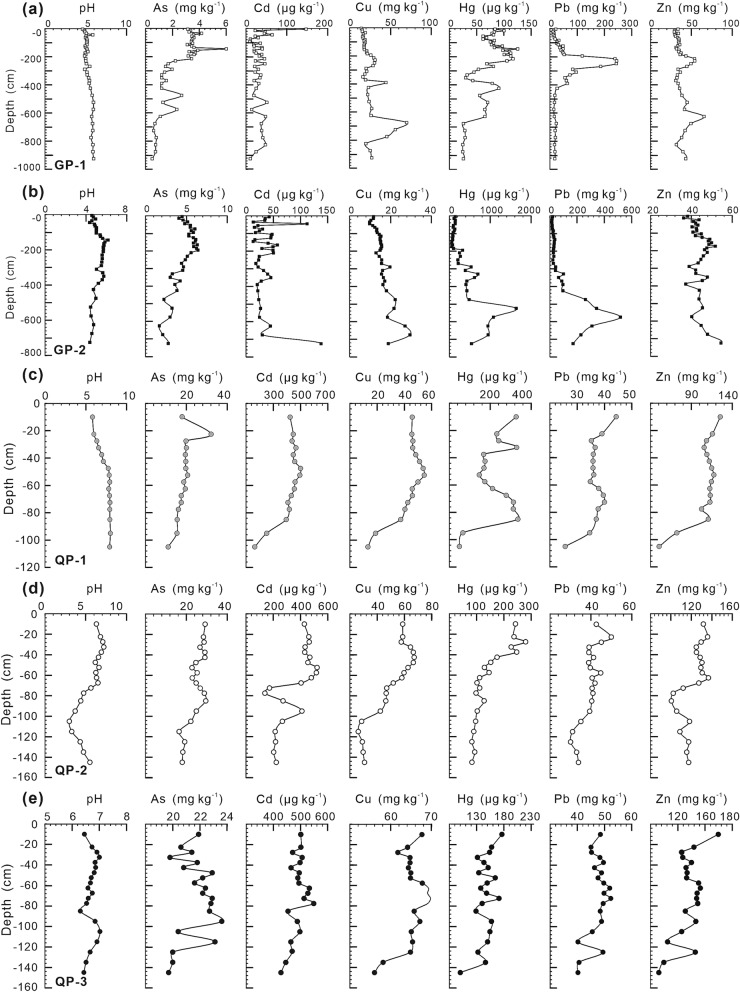
Vertical distributions of pH and potential toxic elements concentrations in the soil profiles developed in granite plutons and quaternary sediments. GP, soil profiles in granite plutons; QP, soil profiles in quaternary sediments.

The medians and ranges of oxides and PTEs in the soil vertical profiles obtained in this investigation can be found as Supplementary Tables S6 and S7 online. The vertical distributions of the PTEs in the soil profiles developed in granite plutons are presented in Fig. 3a,b. The concentration of As is higher in the surface horizons and lower in the deeper horizons. The concentrations of Cd, Cu, Hg, Pb, and Zn vary within a wide range. The vertical distributions of the PTEs in the soil profiles developed in the quaternary sediments are presented in Fig. 3c,d,e. The PTEs exhibit slightly higher concentrations in the surface soils than in the deep soils. The concentrations of PTEs in the soil vertical profiles of the quaternary sediments are significantly higher than those in the soil vertical profiles of granite plutons (see Supplementary Fig. S1 online). The median values of the concentrations of As, Cd, Cu, Hg, Pb, and Zn in the soil vertical profiles of the quaternary sediments are as high as 6.2, 15.2, 3.3, 1.6, 1.4, and 3.0 times, respectively, their median values in the soil vertical profiles of granite plutons.

### Geochemical fractions of Cd and Pb in soil vertical profiles

**Figure 4:**
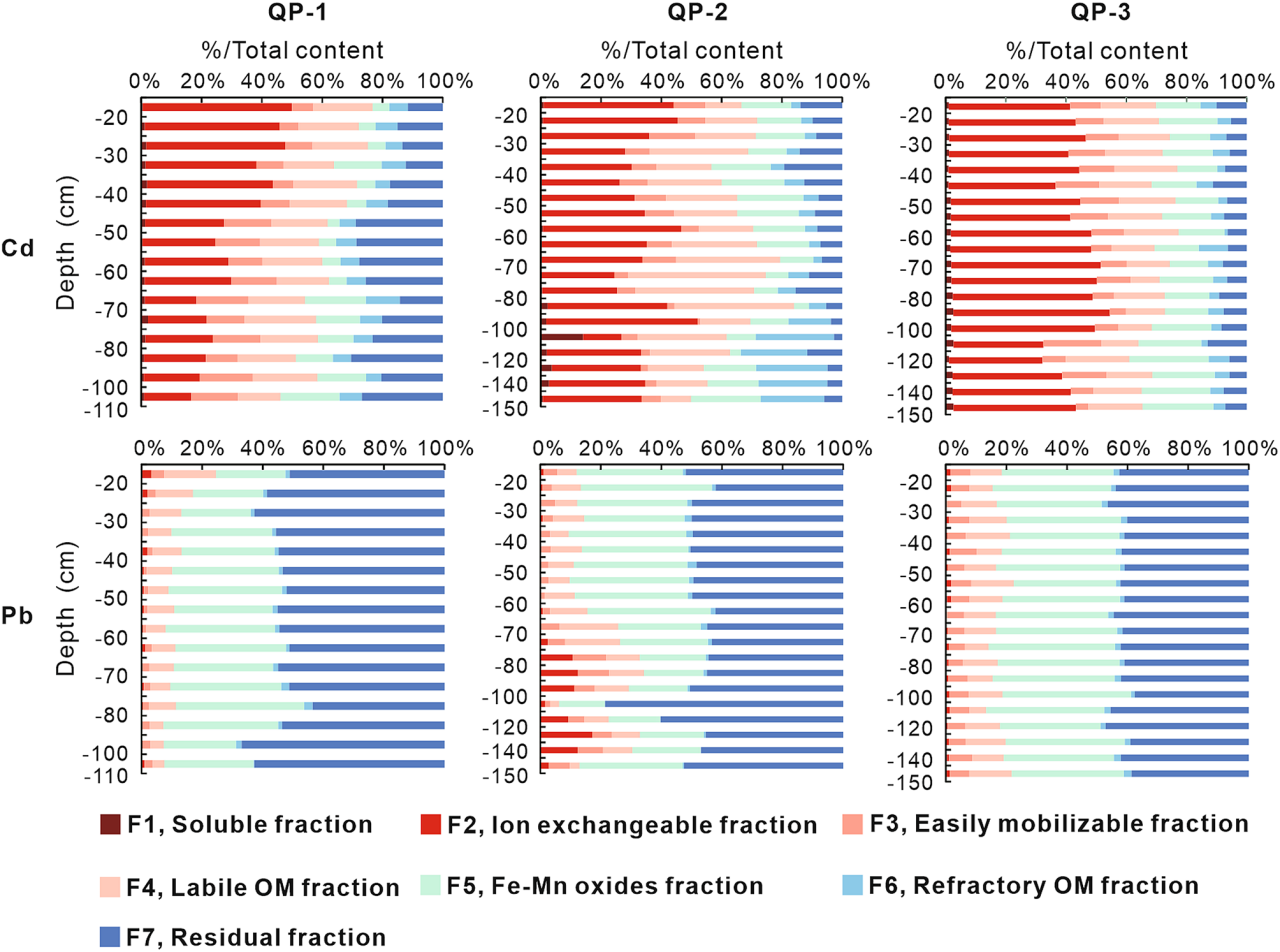
Vertical distributions of Cd and Pb in various geochemical fractions in the soil profiles developed in quaternary sediments.

**Figure 5:**
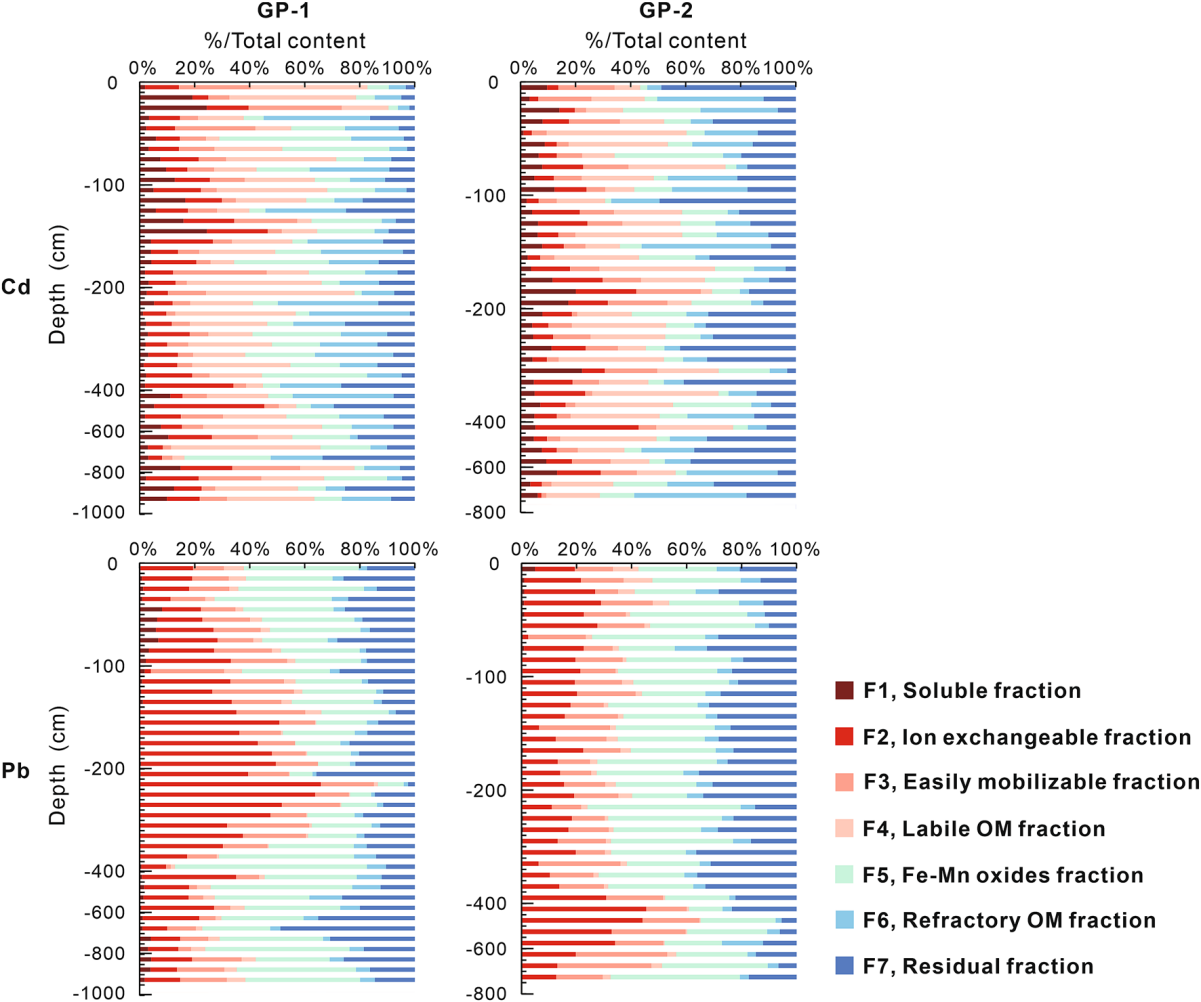
Vertical distributions of Cd and Pb in various geochemical fractions in the soil profiles developed in granite plutons.

The contributions of the different geochemical fractions of Cd and Pb in the soil vertical profiles are depicted in Figs. 4 and 5. The median values of the geochemical fractions of Cd and Pb for each soil profile can be found as Supplementary Table S8 online.

The studied soils demonstrate a large variation in the distribution of Cd among the different geochemical fractions. The relative significance of the Cd geochemical fractions in the soil vertical profiles developed in the quaternary sediments follows the order: F2 > F4 > F5 > F3 ≈ F7 > F6 > F1 (Fig. 4). The relative significance of Cd geochemical fractions in the soil vertical profiles developed in granite plutons is significantly different. It follows the order: F4 > F7 > F6 > F2 ≈ F5 > F3 > F1 (Fig. 5). All Cd geochemical fractions indicate significantly higher median values in the soil vertical profiles developed in quaternary sediments than in those developed in granite plutons (see Supplementary Table S8 online). The mobile fractions (∑F1–F2) of Cd have median values of approximately 119.5–215.7 μg kg^−1^ in the soil vertical profiles developed in quaternary sediments and 3.8–4.4 μg kg^−1^ in the soil vertical profiles developed in granite plutons. Meanwhile, the geochemical fractions of Cd in quaternary boreholes with depths of 23.2–47.0 m in PRD also indicate that Cd has very high mobile fractions^25^. High mobile (and thus ecotoxicologically relevant) amounts of Cd have been demonstrated in other alluvial soils as well^8^. Therefore, Cd poses a potentially higher environmental risk and mobility in soils developed in quaternary sediments than those in granite plutons.

The main geochemical fractions of Pb are residual and Fe–Mn oxides fractions. The percentages of the residual and Fe–Mn oxides fractions in the soil vertical profiles developed in quaternary sediments and granite plutons are 83.30% and 50.59%, respectively. The concentration of Pb in the ion-exchangeable fraction has a higher proportion in the soil vertical profiles developed in granite plutons (19.50%) than those in the soil vertical profiles developed in quaternary sediments (4.89%). The median value of total concentration of Pb in the soil vertical profiles developed in granite plutons (65.99 mg kg^−1^) is higher than in the soil vertical profiles developed in quaternary sediments (41.42 mg kg^−1^). Therefore, Pb in the former has a potentially higher environmental risk and mobility than in the latter.

## Discussion

### Factors affecting distributions of PTEs

**Figure 6:**
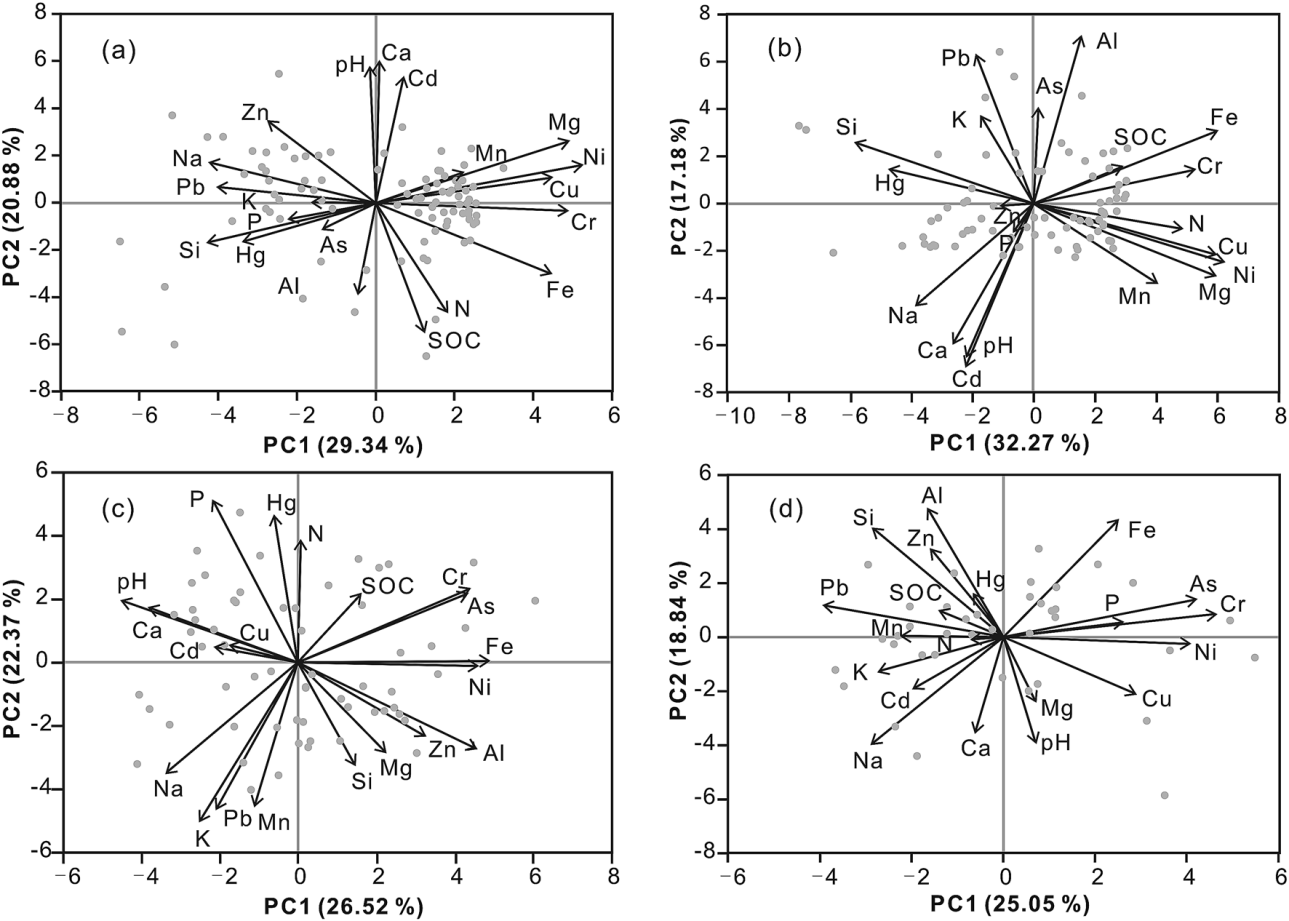
Biplots showing the results of principal components analysis (PCA) for soil geochemical data based on a centered log-ratio transformation (clr). (**a**, **b**), Biplots of PC1 and PC2 for the surface and deep soils developed in quaternary sediments respectively; (**c**, **d**), biplots of PC1 and PC2 for the surface and deep soils developed in granite plutons respectively.

Figure 6a,b depicts biplots (PC1–PC2) for the principal component scores and loadings for the surface and deep soils developed in quaternary sediments. The first three principal components of the surface soils account for 61.3%. Magnesium, Fe, Mn, Ni, Cr, and Cu are grouped along the + PC1 axis. Silicon, Na, K, Pb, P, As, and Hg concentrate along the − PC1 axis. The PC scores along the PC1 axis define a contrast between the mafic (+ scores) and felsic (− scores) source materials. The pH, Ca, and Cd are associated along the + PC2 axis (Fig. 6a). The biplot of Fig. 6b for deep soils depicts similar patterns in terms of the relationships of the elements with each other. The first three principal components for the deep soils account for 60.7%. Magnesium, Fe, Mn, Ni, Cr, and Cu are grouped along the + PC1 axis, probably representing mafic source material (Fig. 6a,b). Silicon, K, Pb, and Hg associations reflect felsic source material. The group of pH, Ca, and Cd probably represents carbonate sources.

Figure 6c, d presents biplots (PC1–PC2) for the principal components scores and loadings for the surface and deep soils developed in granite plutons. The first three principal components account for 61.7% and 60.8%, respectively. The elements of the surface soils concentrate in five groups: (1) Fe, As, Cr, and Ni (+ PC1); (2) SOC, N, P, and Hg (+ PC2); (3) pH, Ca, Cd, and Cu (− PC1); (4) Na, K, Pb, and Mn (− PC1, − PC2); (5) Si, Al, Mg, and Zn (+ PC1, − PC2) (Fig. 6c). The biplot for the deep soils displays different patterns in terms of the relationships of the elements with each other (Fig. 6d). The elements cluster in four groups: (1) Fe, P, As, Cr, Ni, and Cu (+ PC1); (2) Si, Al, Zn (+ PC2, − PC1); (3) K, Na, Cd, and Pb (− PC1), (4) Ca, Mg, and pH (− PC2) (Fig. 6d). For the deep soils, the + PC1 axis in Fig. 6d indicates a chalcophile association of Fe, As, Cr, Ni, and Cu. Cadmium and Pb are grouped with K and Na, probably representing feldspars. For the surface soils, Cd and Cu are clustered with pH and Ca, probably representing pedogenetic processes. The SOC, N, P, and Hg association represents organic materials.

Total concentrations of Cd and Pb and the percentages of their geochemical fractions are correlated with several soil variables in the soil profiles developed in the quaternary sediments (see Supplementary Table S9 online). However, there are almost no correlations among them in the soil profiles developed in granite plutons (see Supplementary Table S10 online). In the soil profiles developed in quaternary sediments, total Cd concentration indicates significant correlations with pH, P, TFe_2_O_3_, CaO, Zn, and Mn. F1 of Cd is negatively correlated with pH. F2 of Cd indicates a strong positive relationship with SOC, N, P, Al_2_O_3_, TFe_2_O_3_, Zn, and the total concentration of Cd. Therefore, the potentially toxicological effect of Cd release is remarkable when the concentrations of nutrient elements (SOC, N, P) in the soils of the study area are high. F3 of Cd is positively correlated with pH and CaO. This fraction of Cd could easily be released with decreasing pH value. The total concentration of Pb indicates significant correlations with the soil variables. F1 of Pb indicates positive relationships with pH, P, and CaO. However, F2 of Pb is negatively correlated with these variables. Therefore, F1 and F2 of Pb could be transformable. F3 of Pb is positively correlated with SOC, whereas it is negatively correlated with pH and Mn. F5 of Pb positively correlates with Mn. Pb may evolve to more crystalline and stable structures of manganese hydrous oxides and become immobile. Alternatively, Pb could be released into the soil solution by the reductive dissolution of manganese oxyhydroxides^8^.

### Major sources of PTEs in deep soils

It is impossible that the features of PTEs in the deep soils are affected by man-made pollution. Therefore, the systematic geochemical differences in PTEs of the deep soils developed between the quaternary sediments and granite plutons suggest that the parent material governs mainly the elemental compositions of the soils.

Other evidences are obtained from the distributions and geochemical fractions of PTEs in the soil vertical profiles or quaternary boreholes. The concentrations of PTEs in the soil vertical profiles developed in quaternary sediments are also significantly higher than in the soil vertical profiles developed in granite plutons (see Supplementary Figure S1 online). There is also a significant difference between the geochemical fractions of Cd and Pb in the soil vertical profiles developed in these two parent materials. The clay layer of a representative quaternary borehole, whose depth is between 12.7 and 26.60 m, has average concentrations of 10.8 mg kg^−1^, 831 μg kg^−1^, and 102 μg kg^−1^ for As, Cd, and Hg, respectively^25^, which are higher than their median values in the deep soils developed in granite plutons (see Supplementary Table S4 online). Comparing these values with those in other alluvial plains of China, the concentrations of PTEs in the deep soils from PRD are significantly higher (see Supplementary Tables S2 and S5 online). Consequently, the PTEs in the deep soils from PRD maybe represent the geochemical characteristics of their parent material. Therefore, we place further emphasis on the major sources of PTEs in the deep soils developed in quaternary sediments.

The PTEs in the deep soils or their parent material were transported by the river water and sediments and originated from various sources in the catchment area at different geological times. The Pb isotopic characteristics of quaternary sediments indicate that the alluvial sediments in PRD had stable sources^26^. The main factors determining the high concentrations of PTEs in the deep soils were source rocks and mineral deposits in the river catchment, particle compositions, and sedimentary environment. The vertical distributions of trace elements in the sediment cores from major rivers in east China indicate that the high concentrations of As, Cd, Hg, Mn, Mo, Pb, and Zn in most southern rivers were predominantly governed by the geological setting, regional mineralization, and geochemical landscape^27^. The concentrations of PTEs in water and suspended particulate matter in the Pearl River and its branches are higher than those in other rivers of China^28^. Numerous carbonate rocks (or karst areas) and super-large or large polymetallic deposits exist in the upper reaches of the West River and North River, which provide large amounts of PTEs for PRD.

Karst areas can affect the concentrations of PTEs in the soils of the Pearl River alluvial plains in the lower reaches in two possible ways. Carbonate rocks are enriched in Cd. Otavite–calcite solid solutions could grow at the (104) cleavage surface of single crystal calcite or dolomite and form Cd-rich carbonate^29,30^. Approximately 5.1 × 10^5^ km^2^ of exposed and outcropped carbonate rock in Southwest China is one of the largest continuous karst areas in the world^31^, and is mainly located in most tributaries of the West River. In the chemical weathering of carbonate rock, Ca, Mg and Cd may be released into the river system, migrating to and depositing in the downstream alluvial plain area. Anomalous Cd and other PTEs have been observed in the soils developed from carbonate rock from a typical karst region in China, which is located in the upper reaches of PRD^32^. The range of concentration of Cd in the soils is 200–5620 μg kg^−1^^32^. Anomalous Cd and other PTEs have also been reported in the soils developed in calcareous rocks from other areas of the world^33–35^. The karst areas of southwest China have experienced severe soil erosion^36,37^. Therefore, soil erosion via riverine inputs could be the other main source of PTEs.

In the natural weathering, mining, and smelting process of polymetallic deposits, a high volume of acid mine drainage rich in PTEs is produced^38^. Several super-large or large polymetallic deposits exit in the upper reaches of PRD, such as the Dabaoshan polymetallic deposit, Fankou Pb–Zn deposit, and Dachang Sn-polymetallic deposit. The chemical weathering of the sulfide minerals in these deposits is very intense owing to abundant rainfall and subtropical climate. For example, the Dabaoshan polymetallic deposit located in the upper reaches of the North River includes stratiform siderite orebodies, gossan formed by weathering processes, stratiform Cu–Pb–Zn orebodies, porphyry-type Mo–W, and skarn-type Mo–W^39^. A large amount of PTEs were released into the environment when the sulfide minerals were oxidized into gossan. Meanwhile, soil erosion in these mine areas has also been very severe^40^. The average values of PTEs in the sediment cores of the North River are the highest among the major rivers in east China^28^. Therefore, the PTEs released by chemical weathering and erosion of polymetallic deposits over time could be transported by the rivers to PRD. These polymetallic deposits have been mined for several years. Therefore, contamination by PTEs in the soils or sediments of these mines is also grave^41–43^. The concentrations of PTEs in the sediments and soils along the river are also very high^44,45^. The PTEs released during the mining and smelting processes could have induced severe surface soil and sediment pollution.

### Major sources of PTEs in surface soils

It is generally believed that the PTEs in the surface soils of economically developed areas are affected by human activities. All the surface soils in this study were collected while avoiding point source pollution. Systematic differences exist in the PTEs between the surface soils developed in the quaternary sediments and granite plutons. The differences are similar to those in the deep soils and the soil vertical profiles (Figs. 1, 2, and 3). Therefore, the parent material is the most significant factor governing the concentrations of PTEs in the surface soils.

The concentrations of As, Cd, Cu, and Zn in the surface soils are higher than in the deep soils developed in quaternary sediments (Fig. 1). The concentration of Hg in the surface soils is higher than that in the deep soils developed in these two parent materials (Fig. 1). Therefore, it appears that human activities have impacted the concentrations of PTEs in the surface soils.

**Figure 7:**
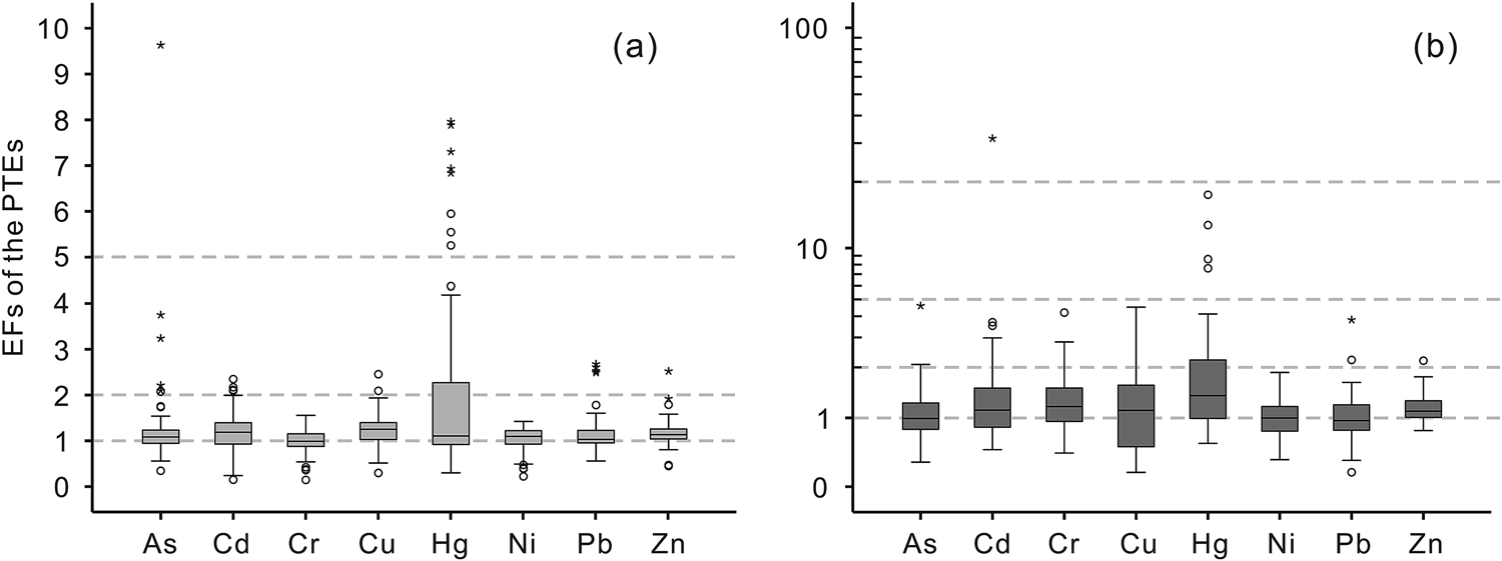
The Tukey boxplots of enrichment factors (EFs) of the potential toxic elements of the surface soils. (**a**) the surface soils developed in quaternary sediments; (**b**) the surface soils developed in granite plutons.

As depicted in Fig. 7, Hg exhibits more significant pollution than the other PTEs. For the surface soils developed in quaternary sediments, the median values of EFs for PTEs are ranked in a decreasing order: Cu (1.25) > Cd (1.19) > Zn (1.13) > Hg (1.11) > Ni (1.10) > As (1.08) > Pb (1.03) > Cr (0.99). Cd, Zn, and Pb are in slight pollution, and Cr and Ni are in slight or no pollution. Some surface soils display As, Cd, Cu, Pb, and Zn in moderate pollution (Fig. 7a). In detail, the EF value of Hg ranges from 0.3 to 8.0, in which approximately 9% of the samples are in significant pollution. For the surface soils developed in granite plutons, the median values of EFs for PTEs are ranked in a decreasing order: Hg (1.50) > Cr (1.24) > Cd, Cu (1.16) > Zn (1.14) > Ni (1.00) > As (0.99) > Pb (0.94). Most surface soils exhibit slight pollution (Fig. 7b). The EF value of Hg ranges from 0.6 to 17.8, with a few samples exceeding 5 (significant pollution). No surface soil samples with the EFs of all the PTEs less than 1 exist, which suggests that anthropogenic activities have significantly impacted the features of the PTEs in the surface soils of PRD. The study of the Pb isotopic and elemental composition of the soils collected from PRD also indicated that Pb in the soils originated from the natural background, automobile exhaust, and industrial pollution^46^.

## Conclusions

In this study, the geochemical distributions and sources of PTEs in the soils developed in the quaternary sediments and granite plutons of PRD were discussed. The concentrations of SOC, oxides and PTEs in the surface soils, deep soils, and soil vertical profiles developed in the quaternary sediments are higher than in the soils developed in granite plutons. Significant differences also exist in the Cd and Pb geochemical fractions and their controlling factors between the soil vertical profiles developed in these two parent materials. These systematic geochemical differences suggest that the parent material predominantly determines the element distributions of the soils. The PTEs of the deep soils developed in quaternary sediments originated mainly from mafic, felsic, and carbonate source materials as well as polymetallic deposits. For the deep soils developed in granite plutons, the elemental associations are governed mainly by their geochemical affinities and behaviors and the mineral compositions of granite plutons. Anthropogenic activities have impacted the features of the PTEs in the surface soils of PRD. However, superimposed regional-scale pollution did not cover the impact of parent material on the distribution of PTEs of the surface soils.

## Materials and methods

### Study area

**Figure 8:**
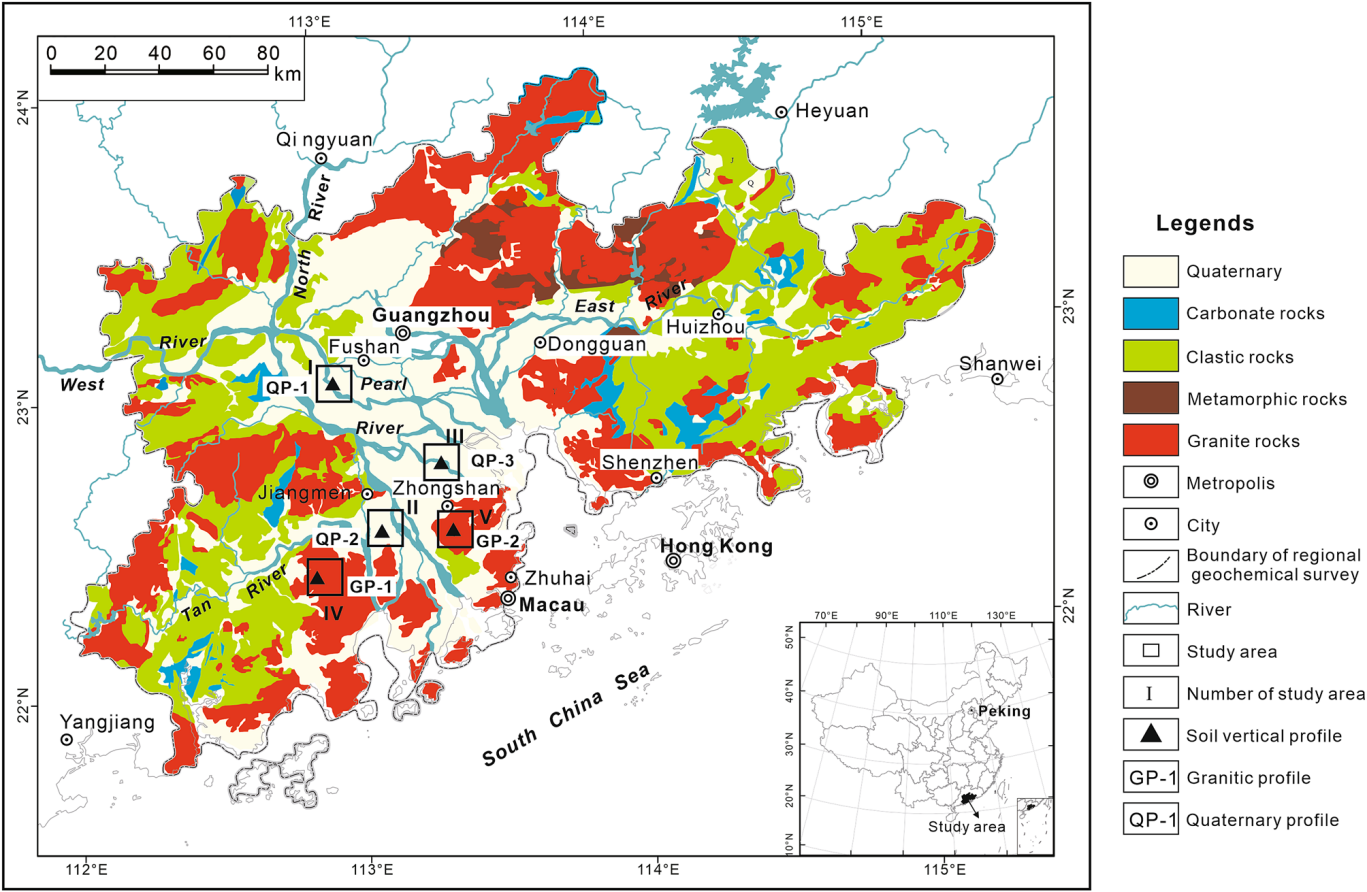
Sampling location and simplified lithological map of Pearl River Delta, South China. Lithological map are modified from^17^. The map was generated with software CorelDRAW Graphics Suite X3 (https://www.corel.com/en/old-versions/coreldraw-x3/).

Pearl River Delta (PRD) is located from 112°00′E to 115°25′E and 22°30′N to 23°45′N, which is an economic zone in the Guangdong Province, South China. PRD covers an area of about 54,764 square kilometers with a population of 59.98 million^47^. It lies within the typical subtropical climate regime with an annual temperature of 21–22 °C and total annual rainfall of 1600–2000 mm. The delta was formed by sediments deposited at the mouth of the Pearl River by the West River, North River, East River, and their tributaries. The shape of the Pearl River delta region is triangular. Approximately, one-fifth of the delta is dotted with granitic plutons and hill platforms composed of metamorphic rocks, sandstones, and shales^48^ (Fig. 8). It includes the big cities of Guangzhou, Zhuhai, Shenzhen, and so on. The sown area of grain crops in PRD is 6453 square kilometers^47^. PRD has undergone rapid industrialization and urbanization during the past three decades. The number of industrial enterprises whose annual principal business revenue are 20 million yuan or above is 32,202^47^. There are several metal smelting and calendering industries, plastic products industries, paper products industries, pharmaceutical manufacturing industries, petroleum processing industries etc^47^. The volume of industrial dust emission and solid wastes produced were 107 thousand tons and 23.95 million tons respectively in 2016^47^.

### Sampling

Surface soils, deep soils and soil vertical profiles were collected from fiver study areas in PRD representing the soils developed in the quaternary sediments and granite plutons respectively (Fig. 8). Study areas numbered I, III and III are located in the distribution area of quaternary sediments. Study areas numbered IV and V are located in the distribution area of granite plutons surrounded by quaternary sediments. Soil vertical profiles numbered QP-1, QP-2 and QP-3 were taken from soils developed in the quaternary sediments, while profiles numbered GP-1 and GP-2 from soils developed in the granite plutons.

Surface soils at depths of 0–20 cm were collected at a density of 1 sample/km^2^ and deep soils (150–180 cm) were collected at a density of 1 sample/4 km^2^. The surface soil samples were composited from 3 to 5 subsamples collected within 50 m of the sampling site. They were never collected from the sites near known point contaminated sources. In addition, no sample was collected at the time of fertilizing. Samples from 4 km^2^ for surface soils and 16 km^2^ for deep soils were composited for chemical analysis. Sites for soil vertical profiles were chosen based on a geologic lithological map in an attempt to represent the parent materials (quaternary sediments and granite plutons). The soil vertical profile samples were collected from pits by avoiding evidently disturbed or contaminated ground. They had no apparent layered structure. Thus, the samples were collected at various intervals. For soil vertical profiles developed in quaternary sediments, the surface soils were collected at depths of 0–20 cm. Other samples were collected at increments of 5 cm from 20 cm up to a depth of 80 cm and at increments of 10 cm at lower depths. For soil vertical profiles developed in granite plutons, samples were taken at increments of 10 cm from 0 m up to a depth of 2 m, at increments 20 cm from 2 m up to a depth of 4 m, and at increments of 50 cm at lower depths.

All the sampling positions were pinpointed by means of the global positioning system (GPS). Visible plant detritus and any rock fragments were removed manually from the soil samples. After air-drying, the samples were crumbled using a wooden hammer, sieved to pass a 20 mesh (< 0.84 mm) Nylon sieve, and further processed with a − 200 mesh (< 0.074 mm) for analysis^49^.

### Chemical analysis

Geochemical data about the surface and deep soils were provided by the Guangdong Geological Bureau, People’s Republic of China. All the data met the chemical analysis quality requirements of the national multipurpose regional geochemical survey in China^17^. The pH, SOC, oxides, and PTEs of the soils in the vertical profiles were analyzed at the Hefei Mineral Resources Supervision and Testing Center, the Ministry of Land and Resources, People’s Republic of China. The samples were pelletized and determined by wavelength dispersive X-ray fluorescence spectrometry PANALITICA AXIOS PW4400 for SiO_2_, Al_2_O_3_, TFe_2_O_3_, MgO, CaO, Na_2_O and K_2_O. Calibrations were conducted using certified reference materials, and *α* correction was applied to correct for matrix interferences. For an analysis of the concentrations of PTEs, soil samples were digested using a mixture of HClO_4_, HNO_3_, and HF in Teflon tubes. The residue was then dissolved with aqua regia, and the supernatant was pipetted and diluted with HNO_3_ (3%). Arsenic and Hg were tested using atomic fluorescence spectrometry (AFS3100, Hai Guang, Inc., China). Cadmium, Cr, Cu, Ni, Pb, and Zn were tested using inductively coupled plasma mass spectrometry (ICAP-7400, Thermo Fisher Scientific, USA). The concentration of SOC was determined by wet oxidation in an acid dichromate solution, followed by back titration of the remaining dichromate using a ferrous ammonium sulfate solution^50^. The soil pH was measured with carbon dioxide—free water (1:2.5, w/w)^51^ using a pH meter (PHS-3CF, Chuangfa, Inc., China). The measurement of nitrogen (N) was conducted with a Perkin Elmer 2400 series II CHNS/O analyzer.

The soil samples were sequentially extracted to determine seven fractions of Cd and Pb^52,53^. Briefly, 2.5 g of soil sample (100 mesh) and 25 mL of extracting agent were shaken for 30 min at 25 °C. The samples were centrifuged at 4000×*g* for 20 min and filtered. The extracting agents and their operationally defined fractions can be found as Supplementary Table S11 online. The elements were sequentially fractionated into seven fractions as follows: F1—soluble fraction, F2—ion exchangeable fraction, F3—easily mobilizable fraction, F4—labile organic matter (OM) fraction, F5—Fe–Mn oxides fraction, F6—refractory OM fraction, and F7—residual fraction.

The element concentrations in the digests and extracts were measured using inductively coupled plasma-atomic emission spectroscopy (ICP-AES) (X-SERIES II, Thermo Fisher Scientific, USA). Total digestion was performed on the samples as an internal check.

### Quality control

Standard reference and replicate samples were used to monitor the trueness, repeatability, accuracy, and precision. The detection limits and allowance of accuracy and precision for routine analysis were discussed in details^49^. The analytical accuracy of the concentrations of the soil elements was controlled by the national standard material GSS02-GSS26. Coded samples were added to control the precision of the analytical data. The accuracy and precision of the elemental analyses of all samples met the quality requirements. To guarantee very high quality of the achieved results, the sum of each element extracted in the seven-step sequential extraction (ΣF1–F7) was compared with the results obtained by a separate aqua regia digestion as follows:

$${\text{Recovery}} = \frac{{{\text{C}}_{{{\text{sum}}}} - {\text{C}}_{{{\text{total}}}} }}{{{\text{C}}_{{{\text{total}}}} }} \times 100\%$$

where C_sum_ is the sum of each element extracted in the seven-step sequential extraction and C_total_ is the concentration obtained by a separate aqua regia digestion.

In all measurements, the concentrations of the elements in the extracts were measured eight times, and analyses of standard reference samples were routinely included for quality control. The considered soil geochemical fractions of element results represent the arithmetic means of duplicate samples. The maximum relative standard deviation between replicates was less than 20%.

### Data processing

Geochemical data are a classic example of compositional (closed) data, and the element concentrations depend on each other^54,55^. This is significant for correlation analysis, for which the data must be appropriately log-ratio transformed^56^. The concentrations of the soil elements were adjusted using a centered log-ratio (clr) transformation^56–58^, which overcomes the “closure problem” produced by compositional data.

The *clr* transformation is defined as:

$${\text{clr}}\left( {\varvec{x}} \right) = \left[ {{\ln}\frac{{x_{1} }}{{g\left( {\varvec{x}} \right)}}{\ln}\frac{{x_{2} }}{{g\left( {\varvec{x}} \right)}} \ldots {\ln}\frac{{x_{D} }}{{g\left( {\varvec{x}} \right)}}{ }} \right],$$

where ${\text{g}}\left( x \right) = \left[ {x_{1} \cdot x_{2} \ldots \cdot x_{D} } \right]^{1/D}$ is the geometric mean of the composition ***x***.

Robust compositional biplots were generated to better visualize the element distributions and quantify the main principal components. The scores represent the structure of the compositional data in the Euclidian space based on the variance and covariance matrices. Moreover, they display the association structure of the dataset. The variables are represented by rays (or vectors) drawn from the center of the plot, whose lengths are proportional to the amount of their explained variance (communality). The interpretation of the plots depends on the loading (the length of rays) structures and, in further details, on the approximate links between the rays and samples and the distances between the vertices and their directions^59^.

The median values of PTEs in the deep soils from PRD are considered as local background values, we can use the enrichment factor (EF) to quantify the extent of pollution caused by PTEs in the surface soils. The EF is calculated as follows:

$${\text{EF}} = \frac{{\left[ {{\text{x}}/{\text{R}}} \right]_{{{\text{sample}}}} }}{{\left[ {{\text{x}}/{\text{R}}} \right]_{{{\text{background}}}} }}$$

where [*x*/*R*]_sample_ and [*x*/*R*]_background_ are the concentration ratios of the PTE and normalizer in the surface and deep soils, respectively. The conservative elements widely used for normalization include Ti, Al, Fe, Co, and Sc^60,61^, and Al was selected as the reference in this study. The median values of the concentrations of elements in deep soils (see Supplementary Tables S2 and S4 online) were used as the normalizers. The EF value is always classified into several grades, as follows: EF < 1, no pollution; 1 < EF < 2, slight pollution; 2 < EF < 5, moderate pollution; 5 < EF < 20, significant pollution; EF > 20, severe pollution^62^.

## Supplementary information

Supplementary Information.
