# Supplementary material for: Impacts of parent material on distributions of potentially toxic elements in soils from Pearl River Delta in South China
Source: Sci Rep. 2020 Oct 15;10:17394. doi: 10.1038/s41598-020-74490-2 (PMC7567083; doi:10.1038/s41598-020-74490-2)
Supplement: Supplementary file 1 — Supplementary Information. [file 41598_2020_74490_MOESM1_ESM.pdf]

Impacts of parent material on distributions of potentially toxic elements in soils from Pearl River Delta in South China

Qingye Hou, Zhongfang Yang, Tao Yu, Yuanhang You, Lei Dou , Kuo Li

Parameter	Units	n	Median	Extreme values		Centile		Quartile		Not-transformed		Ln-transformed	
				Min	Max	5th	95th	1st	3rd	Skewness	Kurtosis	Skewness	Kurtosis
pH		90	6.40	4.26	8.32	4.82	7.86	5.81	7.17	-0.13	-0.66	-0.44	-0.37
SOC	%	90	1.32	0.35	2.15	0.60	1.88	0.98	1.54	-0.20	-0.26	-1.22	2.05
SiO_2	%	90	62.57	57.01	86.44	58.02	76.77	59.26	69.75	0.88	0.10	0.71	-0.43
Al_2O_3	%	90	15.94	5.52	25.22	9.24	18.32	13.56	17.39	-0.65	1.66	-1.70	4.20
TFe_2O_3	%	90	7.02	2.61	8.72	3.61	8.48	4.40	7.96	-0.35	-1.44	-0.61	-0.95
MgO	%	90	1.15	0.16	1.57	0.44	1.40	0.74	1.29	-0.58	-0.68	-1.65	3.50
CaO	%	90	0.61	0.08	1.41	0.19	1.26	0.47	0.81	0.55	0.32	-1.22	2.59
Na_2O	%	90	0.37	0.05	0.89	0.28	0.49	0.35	0.40	1.70	11.05	-3.43	23.58
K_2O	%	90	2.42	0.76	4.22	1.68	2.95	2.21	2.53	0.31	6.56	-2.13	12.06
As	mg kg^{-1}	90	18.5	2.4	100.1	9.6	29.9	13.1	21.8	4.53	28.73	-0.08	5.29
Cd	μg kg^{-1}	90	458	68	762	116	666	356	518	-0.51	0.51	-1.94	4.05
Cr	mg kg^{-1}	90	87.8	20.0	122.7	43.0	117.4	60.0	98.0	-0.35	-0.76	-1.17	1.55
Cu	mg kg^{-1}	90	58.6	14.3	118.3	25.0	75.6	37.5	65.1	0.25	0.64	-0.73	0.13
Hg	μg kg^{-1}	90	141	36	803	74	711	122	260	2.01	3.20	0.68	0.61
Mn	mg kg^{-1}	90	751	75	1146	373	965	484	851	-0.35	-0.60	-1.97	7.19
N	mg kg^{-1}	90	1424	100	2648	409	2313	964	1785	0.01	-0.62	-1.48	3.28
Ni	mg kg^{-1}	90	38.4	5.8	53.7	13.8	50.0	24.2	46.5	-0.39	-1.07	-1.23	1.68
P	mg kg^{-1}	90	818	182	1449	432	1170	691	933	0.05	1.07	-1.57	5.33
Pb	mg kg^{-1}	90	42.0	11.3	102.7	29.0	66.2	38.1	45.0	2.58	9.28	-0.21	8.04
Zn	mg kg^{-1}	90	125.2	21.2	192.0	80.0	148.8	101.4	136.8	-0.84	2.06	-2.96	14.82

Supplementary Table S1 Statistical summary of chemical analyses of the surface soils developed in quaternary sediments from PRD, South China.

Parameter	Units	n	Median	Extreme values		Centile		Quartile		Not-transformed		Ln-transformed	
				Min	Max	5th	95th	1st	3rd	Skewness	Kurtosis	Skewness	Kurtosis
pH		79	7.33	3.98	8.19	4.76	7.93	6.71	7.57	-1.52	2.08	-1.91	3.69
SOC	%	79	0.95	0.48	1.73	0.54	1.45	0.80	1.13	0.52	0.36	-0.23	-0.23
SiO_2	%	79	63.68	56.63	79.96	57.33	78.79	58.57	70.20	0.68	-0.57	0.54	-0.83
Al_2O_3	%	79	16.52	8.85	23.33	9.63	19.07	13.85	18.11	-0.40	-0.15	-0.89	0.34
TFe_2O_3	%	79	6.77	2.91	8.84	3.81	8.74	5.17	7.99	-0.36	-1.00	-0.76	-0.18
MgO	%	79	1.09	0.35	1.55	0.53	1.53	0.86	1.35	-0.38	-0.78	-1.03	0.80
CaO	%	79	0.72	0.29	1.62	0.33	1.13	0.53	0.87	0.62	1.13	-0.45	-0.04
Na_2O	%	79	0.36	0.17	0.46	0.23	0.41	0.33	0.38	-1.39	2.34	-2.04	4.85
K_2O	%	79	2.34	1.32	3.58	1.66	2.76	2.07	2.55	0.03	1.41	-0.66	1.32
As	mg kg^{-1}	79	17.1	8.5	57.6	9.1	22.7	13.2	19.9	3.46	22.32	0.29	2.54
Cd	μg kg^{-1}	79	405	132	1245	182	587	338	479	2.55	15.59	-0.59	3.45
Cr	mg kg^{-1}	79	89.0	43.0	117.0	50.0	114.0	74.0	107.0	-0.40	-0.85	-0.84	0.06
Cu	mg kg^{-1}	79	46.3	14.4	75.8	24.3	64.4	36.4	56.9	-0.20	-0.65	-0.94	0.95
Hg	μg kg^{-1}	79	134	38	1366	72	414	116	180	4.67	25.61	1.40	4.23
Mn	mg kg^{-1}	79	733	217	1137	272	1039	519	890	-0.20	-0.85	-0.90	0.26
N	mg kg^{-1}	79	1092	302	1987	499	1714	845	1325	0.08	-0.31	-0.91	1.13
Ni	mg kg^{-1}	79	35.9	10.7	51.2	19.5	48.8	29.1	44.5	-0.39	-0.73	-1.13	1.66
P	mg kg^{-1}	79	563	313	995	365	824	480	636	0.74	1.62	-0.12	0.33
Pb	mg kg^{-1}	79	42.1	29.6	156.7	31.4	60.2	35.9	47.7	4.63	24.39	2.49	9.95
Zn	mg kg^{-1}	79	114.0	58.0	195.0	74.8	142.0	94.0	129.0	0.17	1.02	-0.55	0.43

Supplementary Table S2 Statistical summary of chemical analyses of the deep soils developed in quaternary sediments from PRD, South China.

Parameter	Units	n	Median	Extreme values		Centile		Quartile		Not-transformed		Ln-transformed	
				Min	Max	5th	95th	1st	3rd	Skewness	Kurtosis	Skewness	Kurtosis
pH		60	4.98	4.48	7.70	4.62	6.22	4.80	5.22	0.01	-1.13	-0.03	-1.11
SOC	%	60	0.89	0.36	1.69	0.46	1.52	0.74	1.14	0.04	-0.78	-0.40	-0.66
SiO_2	%	60	62.27	50.51	83.58	55.03	76.33	59.53	65.64	0.09	-0.69	0.01	-0.70
Al_2O_3	%	60	26.84	7.66	34.50	14.42	31.46	21.38	28.62	-0.05	-0.54	-0.21	-0.48
TFe_2O_3	%	60	3.11	0.83	6.18	1.86	5.56	2.62	4.10	0.42	-1.00	0.10	-1.07
MgO	%	60	0.11	0.06	0.25	0.07	0.22	0.09	0.13	-0.03	-0.71	-0.37	-0.59
CaO	%	60	0.08	0.03	0.82	0.04	0.38	0.05	0.14	0.24	-0.58	-0.24	-0.65
Na_2O	%	60	0.11	0.02	0.33	0.03	0.26	0.05	0.16	0.27	-1.28	-0.38	-1.16
K_2O	%	60	1.47	0.31	4.18	0.39	3.58	0.97	1.88	-0.28	-0.81	-1.09	0.39
As	mg kg^{-1}	60	3.7	1.1	17.3	1.8	8.3	2.8	4.6	-0.24	-1.00	-0.66	-0.57
Cd	μg kg^{-1}	60	60	36	186	38	167	51	85	0.21	-0.94	-0.16	-0.87
Cr	mg kg^{-1}	60	15.3	5.0	70.0	7.0	38.0	12.0	18.0	-0.29	-0.79	-0.69	-0.47
Cu	mg kg^{-1}	60	4.7	0.8	22.0	1.0	14.7	2.6	8.3	0.39	-1.07	-0.53	-0.62
Hg	μg kg^{-1}	60	76	28	558	39	237	54	123	0.27	-0.95	-0.29	-0.55
Mn	mg kg^{-1}	60	213	116	385	135	377	185	277	0.41	-0.68	0.05	-0.68
N	mg kg^{-1}	60	782	229	2143	259	1385	550	1027	-0.06	-0.79	-0.91	0.34
Ni	mg kg^{-1}	60	5.0	2.0	13.0	2.2	10.8	3.8	6.6	0.01	-0.63	-0.70	0.02
P	mg kg^{-1}	60	204	77	1270	90	1047	123	509	-0.09	-1.01	-0.20	-1.00
Pb	mg kg^{-1}	60	44.0	10.0	130.0	19.3	78.5	36.1	54.1	-0.14	-0.43	-0.80	0.22
Zn	mg kg^{-1}	60	44.9	22.0	84.0	34.0	63.5	39.3	51.1	0.22	-0.83	-0.04	-0.86

Supplementary Table S3 Statistical summary of chemical analyses of the surface soils developed in granite plutons from PRD, South China.

Parameter	Units	*n*	Median	Extreme values		Centile		Quartile		Not-transformed		Ln-transformed	
				Min	Max	5th	95th	1st	3rd	Skewness	Kurtosis	Skewness	Kurtosis
pH		38	4.97	4.71	6.63	4.75	5.83	4.85	5.23	2.45	8.06	2.13	6.13
SOC	%	38	0.34	0.18	0.71	0.19	0.67	0.28	0.46	0.82	0.36	0.05	-0.56
SiO_2	%	38	58.30	47.04	68.71	50.13	68.12	55.02	61.61	0.01	-0.48	-0.20	-0.31
Al_2O_3	%	38	29.01	20.21	35.60	20.71	34.65	25.02	30.26	-0.18	-0.46	-0.49	-0.27
TFe_2O_3	%	38	3.81	2.32	8.44	2.36	7.80	3.04	5.26	0.98	0.18	0.40	-0.69
MgO	%	38	0.13	0.05	0.76	0.07	0.31	0.09	0.17	4.08	20.55	1.06	3.05
CaO	%	38	0.06	0.04	0.53	0.04	0.39	0.05	0.10	2.92	9.32	1.43	1.44
Na_2O	%	38	0.05	0.02	0.29	0.02	0.28	0.03	0.09	1.80	2.43	0.68	-0.34
K_2O	%	38	1.12	0.29	3.50	0.31	3.38	0.70	1.92	1.00	0.41	-0.22	-0.55
As	mg kg^{-1}	38	4.3	2.0	19.1	2.6	15.5	3.3	6.4	2.40	6.87	0.85	0.76
Cd	μg kg^{-1}	38	67	27	269	29	139	51	79	3.49	16.69	0.54	2.47
Cr	mg kg^{-1}	38	15.0	5.0	89.0	6.0	58.0	11.0	22.0	2.69	8.96	0.55	0.61
Cu	mg kg^{-1}	38	5.5	0.6	29.8	2.1	22.5	3.9	8.3	1.93	3.56	-0.17	1.67
Hg	μg kg^{-1}	38	57	26	320	34	154	46	82	3.82	18.24	1.27	3.05
Mn	mg kg^{-1}	38	200	82	484	113	452	166	271	1.15	1.06	0.17	0.02
N	mg kg^{-1}	38	274	125	656	161	577	212	368	1.20	1.74	0.20	-0.06
Ni	mg kg^{-1}	38	6.2	3.6	41.8	3.6	24.8	5.2	7.8	3.43	13.28	1.83	3.64
P	mg kg^{-1}	38	145	78	456	78	447	104	282	0.97	-0.32	0.40	-1.18
Pb	mg kg^{-1}	38	53.8	16.8	134.2	20.6	97.7	33.9	75.9	0.67	0.45	-0.37	-0.46
Zn	mg kg^{-1}	38	45.0	28.0	93.0	32.0	81.0	40.0	51.0	2.01	6.23	0.82	2.38

Supplementary Table S4 Statistical summary of chemical analyses of the deep soils developed in granite plutons from PRD, South China.

Elements	Units	upper continental crust (UCC)	background values of China soils (BVCS)	deep soils of alluvial plain in theYangtze River Delta	deep soils of alluvial plain in theYellow River Delta	deep soils of alluvial plain in the Haihe River Delta
As	mg kg^{-1}	4.8	9.2	9.29	9.7	9.42
Cd	μg kg^{-1}	90	74	90	103	100
Cr	mg kg^{-1}	92	53.9	72.4	62.7	69.11
Cu	mg kg^{-1}	28	20	22.5	20.3	22.85
Hg	μg kg^{-1}	50	40	40	16	16.32
Ni	mg kg^{-1}	47	23.4	30	26.4	29.72
Pb	mg kg^{-1}	17	23.6	24.3	18.3	21
Zn	mg kg^{-1}	67	67.7	68	57.1	63.92
Data sources		19	20	22	23	24

Supplementary Table S5 Concentrations of potential toxic elements in upper continental crust, and in the soils of major alluvial plain of China, and the background values of China soils.

Parameter	Units	n	Median	Extreme values		Centile		Quartile		Not-transformed		Ln-transformed	
				Min	Max	5th	95th	1st	3rd	Skewness	Kurtosis	Skewness	Kurtosis
pH		56	6.67	2.98	8.00	3.70	7.95	6.30	7.01	-1.28	1.87	-1.94	4.09
SOC	%	56	1.17	0.12	1.83	0.36	1.77	0.56	1.49	-0.24	-1.27	-1.10	0.94
SiO_2	%	56	60.54	58.45	80.12	58.58	67.76	59.71	63.11	2.94	11.55	2.60	9.22
Al_2O_3	%	56	17.68	8.20	18.61	14.48	18.39	16.85	17.91	-3.43	14.63	-4.28	21.96
TFe_2O_3	%	56	7.94	3.30	9.59	5.58	9.21	6.74	8.13	-1.17	2.35	-2.10	6.76
MgO	%	56	1.29	0.51	1.52	1.02	1.42	1.20	1.32	-2.51	9.93	-3.76	18.34
CaO	%	56	0.64	0.32	1.03	0.34	0.79	0.52	0.69	-0.12	1.02	-0.91	0.71
Na_2O	%	56	0.33	0.31	0.47	0.31	0.42	0.32	0.37	1.39	1.92	1.13	0.91
K_2O	%	56	2.55	2.22	3.39	2.30	2.69	2.46	2.60	2.44	13.75	1.69	9.46
As	mg kg^{-1}	56	21.7	11.1	32.1	15.5	29.2	19.6	24.2	0.28	0.17	-0.44	1.32
Cd	μg kg^{-1}	56	455	138	548	173	526	414	494	-1.44	0.94	-1.83	2.32
Cr	mg kg^{-1}	56	117.2	91.3	320.9	96.4	200.4	111.2	126.4	3.28	12.86	2.21	6.18
Cu	mg kg^{-1}	56	57.9	13.2	69.7	26.1	69.0	45.8	64.9	-0.96	0.26	-1.88	4.15
Hg	μg kg^{-1}	56	151	49	335	83	327	128	194	0.91	0.20	-0.10	0.13
Mn	mg kg^{-1}	56	860	276	1245	318	1190	667	931	-0.56	-0.36	-1.18	0.34
N	mg kg^{-1}	56	1116	260	2026	507	1985	808	1657	0.12	-1.12	-0.73	0.43
Ni	mg kg^{-1}	56	43.8	16.4	54.0	32.3	52.1	37.6	50.0	-1.09	1.61	-2.12	6.66
P	mg kg^{-1}	56	693	302	907	317	888	572	819	-0.67	-0.54	-1.13	0.28
Pb	mg kg^{-1}	56	40.5	25.6	52.3	31.0	50.0	36.8	47.0	-0.12	-0.52	-0.51	0.30
Zn	mg kg^{-1}	56	126.7	50.6	169.7	100.5	155.2	112.7	144.6	-0.73	1.80	-1.95	7.36

Supplementary Table S6 Statistical summary of chemical analyses in the soil vertical profiles developed in quaternary sediments from PRD, South China.

Parameter	Units	*n*	Median	Extreme values		Centile		Quartile		Not-transformed		Ln-transformed	
				Min	Max	5th	95th	1st	3rd	Skewness	Kurtosis	Skewness	Kurtosis
pH		78	5.14	4.37	6.16	4.51	5.92	4.95	5.64	-0.96	0.04	-0.87	-0.10
SOC	%	78	0.16	0.04	2.00	0.05	1.27	0.10	0.29	8.12	2.87	0.76	0.98
Al_2O_3	%	78	31.68	27.54	33.83	28.16	33.53	30.31	32.70	-0.58	-0.60	-0.47	-0.68
MgO	%	78	0.21	0.07	0.46	0.09	0.40	0.13	0.27	-0.45	0.69	-1.14	0.03
CaO	%	78	0.07	0.04	0.25	0.04	0.18	0.05	0.11	0.07	1.04	-1.12	0.51
Na_2O	%	78	0.05	0.04	0.09	0.04	0.09	0.05	0.06	0.84	1.29	0.16	0.92
K_2O	%	78	0.79	0.35	3.74	0.38	3.40	0.42	1.34	0.67	1.37	-0.97	0.59
As	mg kg^{-1}	78	3.5	0.5	6.4	0.7	6.2	2.2	4.9	-0.99	-0.06	0.33	-1.06
Cd	μg kg^{-1}	78	30	9	147	11	64	22	45	11.38	2.98	1.01	0.37
Cr	mg kg^{-1}	78	166.1	82.8	666.0	89.0	466.8	113.8	235.0	4.13	1.86	-0.26	0.58
Cu	mg kg^{-1}	78	17.8	9.8	69.7	11.3	44.5	15.3	21.7	10.99	2.92	2.45	1.20
Hg	μg kg^{-1}	78	93	25	1635	26	943	68	144	11.02	3.08	0.40	0.84
N	mg kg^{-1}	78	246	169	1444	184	983	215	353	9.73	2.98	2.24	1.58
Ni	mg kg^{-1}	78	7.1	1.0	11.0	1.5	10.2	3.5	8.4	-1.18	-0.37	-0.32	-0.99
P	mg kg^{-1}	78	125	72	311	80	166	100	139	11.04	2.15	1.50	0.37
Pb	mg kg^{-1}	78	29.3	11.5	516.9	13.6	260.3	18.9	63.6	8.79	2.79	0.17	1.02
Zn	mg kg^{-1}	78	41.8	29.0	65.5	30.9	54.0	34.8	45.3	0.61	0.55	-0.39	0.09

Supplementary Table S7 Statistical summary of chemical analyses in the soil vertical profiles developed in granite plutons from PRD, South China.

	F1	F2	F3	F4	F5	F6	F7	∑F1-F2	∑F1-F6	∑F1-F7
**Cd (**µg kg^{-1}**)**										
QP-1	5.0	114.5	40.0	77.0	25.8	25.8	72.9	119.5	316.0	407.5
QP-2	1.5	127.0	33.7	81.0	53.4	20.4	30.6	128.5	369.7	414.8
QP-3	8.7	207.0	51.9	80.6	80.0	22.7	33.8	215.7	451.0	490.0
GP-1	1.0	3.4	1.7	6.0	2.6	4.2	2.7	4.4	21.2	26.1
GP-2	2.0	1.8	2.0	6.4	2.0	3.0	5.5	3.8	21.0	28.5
**Pb (**mg kg^{-1}**)**										
QP-1	0.06	0.14	0.68	2.51	10.42	0.43	17.25	0.20	13.90	31.21
QP-2	0.02	0.42	1.73	3.68	12.38	0.49	18.97	0.43	20.11	39.78
QP-3	0.09	0.32	2.78	4.86	17.37	0.83	18.88	0.41	26.13	44.79
GP-1	0.27	6.92	5.25	0.74	8.06	1.05	5.27	7.19	21.73	26.33
GP-2	0.10	4.61	3.97	0.81	9.01	1.49	7.49	4.70	20.02	26.52

GP, soil profiles developed in granite pluton.

QP, soil profiles developed in quaternary sediments.

Supplementary Table S8 Median values of individual geochemical fractions of Cd and Pb in soil vertical profiles from PRD, South China.

	Total	F1	F2	F3	F4	F5	F6	F7
**Cd**								
pH	0.49 [a]	-0.49	n.s	0.77 [a]	-0.32 [b]	n.s	-0.65 [a]	0.59 [a]
SOC	n.s	n.s	0.48 [a]	-0.29 [b]	n.s	n.s	n.s	-0.70 [a]
N	0.41 [a]	n.s	0.67 [a]	n.s	n.s	n.s	n.s	-0.64 [a]
P	0.80 [a]	-0.28 [b]	0.49 [a]	0.43 [a]	-0.42 [a]	n.s	-0.70 [a]	n.s
SiO_2	-0.55 [a]	n.s	-0.47 [a]	n.s	n.s	n.s	n.s	0.53 [a]
Al_2O_3	0.33 [b]	n.s	0.37 [a]	-0.36 [a]	n.s	n.s	n.s	-0.59 [a]
TFe_2O_3	0.57 [a]	n.s	0.37 [a]	n.s	n.s	n.s	-0.43 [a]	-0.40 [a]
MgO	0.32 [b]	n.s	0.29 [b]	-0.29 [b]	n.s	n.s	n.s	-0.46 [a]
CaO	0.68 [a]	n.s	n.s	0.55 [a]	-0.46 [a]	n.s	-0.37 [a]	0.33 [b]
K_2O	-0.38 [a]	n.s	n.s	n.s	n.s	n.s	n.s	-0.29 [b]
Na_2O	-0.52 [a]	n.s	-0.37 [a]	n.s	n.s	-0.29 [b]	0.46 [a]	0.44 [a]
Zn	0.69 [a]	n.s	0.60 [a]	n.s	-0.29 [b]	0.29 [b]	-0.30 [b]	-0.54 [a]
Mn	0.79 [a]	-0.36 [a]	n.s	0.44 [a]	n.s	n.s	-0.61 [a]	n.s
Total	1	n.s	0.51 [a]	0.33 [b]	-0.45 [a]	n.s	-0.61 [a]	n.s
**Pb**								
pH	n.s	0.34 [b]	-0.72 [a]	-0.45 [a]	n.s	0.67 [a]	0.54 [a]	n.s
SOC	0.68[a]	n.s	n.s	0.63 [a]	0.30 [b]	n.s	n.s	-0.48 [a]
N	0.88 [a]	n.s	n.s	0.53 [a]	0.33 [b]	0.34 [a]	n.s	-0.61 [a]
P	0.75 [a]	0.42 [a]	-0.59 [a]	n.s	n.s	0.71 [a]	0.51 [a]	-0.47 [a]
SiO_2	-0.63 [a]	n.s	n.s	-0.30 [b]	-0.32 [b]	-0.38 [a]	-0.29 [b]	0.56 [a]
Al_2O_3	0.43 [a]	n.s	n.s	0.39 [a]	0.27 [b]	n.s	n.s	-0.49 [a]
TFe_2O_3	0.61 [a]	n.s	n.s	n.s	0.30 [b]	0.49 [a]	0.38 [a]	-0.54 [a]

MgO	0.29 [b]	n.s	n.s	0.26 [b]	n.s	n.s	n.s	-0.43 [a]
CaO	0.31 [b]	0.35 [a]	-0.51 [a]	-0.29 [b]	n.s	0.64 [a]	0.41 [a]	n.s
K_2O	n.s	n.s	n.s	0.37 [a]	n.s	n.s	n.s	n.s
Na_2O	-0.67 [a]	n.s	n.s	-0.32 [b]	-0.35 [a]	-0.51 [a]	-0.41 [a]	0.67 [a]
Zn	0.84 [a]	n.s	n.s	0.32 [b]	0.32 [b]	0.56 [a]	0.34 [b]	-0.67 [a]
Mn	0.33 [b]	n.s	-0.69 [a]	-0.52 [a]	n.s	0.68 [a]	0.51 [a]	n.s
Total	1	n.s	-0.31 [b]	0.37 [a]	0.41 [a]	0.55 [a]	0.40 [a]	-0.69 [a]

n.s: not significant.

[a] Correlation is significant at 0.05 level.

[b] Correlation is significant at 0.01 level.

Supplementary Table S9 Pearson correlation coefficients between relevant soil properties and Cd and Pb geochemical fractions (% of total) in the soil vertical profiles developed in quaternary sediments.

	Total	F1	F2	F3	F4	F5	F6	F7
**Cd**								
pH	n.s	n.s	n.s	n.s	n.s	n.s	n.s	n.s
SOC	n.s	n.s	-0.25 [b]	n.s	n.s	n.s	n.s	n.s
Al_2O_3	n.s	n.s	n.s	n.s	n.s	n.s	n.s	n.s
MgO	n.s	n.s	0.24 [b]	n.s	n.s	n.s	n.s	-0.29 [b]
CaO	n.s	n.s	n.s	n.s	n.s	n.s	-0.22 [b]	n.s
Na_2O	n.s	n.s	n.s	n.s	n.s	n.s	n.s	n.s
K_2O	n.s	n.s	0.28 [b]	n.s	n.s	n.s	n.s	n.s
P	n.s	n.s	n.s	n.s	0.23 [b]	n.s	n.s	-0.27 [b]
Total	1	n.s	n.s	n.s	n.s	n.s	n.s	n.s
**Pb**								
pH	-0.39[a]	n.s	-0.37 [a]	-0.29 [a]	n.s	0.23 [b]	n.s	0.43 [a]
SOC	n.s	n.s	n.s	n.s	0.50 [a]	n.s	n.s	n.s
Al_2O_3	n.s	-0.33 [a]	n.s	0.26 [b]	-0.41 [a]	-0.27 [b]	n.s	0.22 [b]
MgO	-0.23 [b]	0.30 [a]	n.s	-0.34 [a]	n.s	n.s	n.s	n.s
CaO	-0.30 [a]	n.s	-0.50 [a]	n.s	n.s	0.28 [b]	n.s	0.44 [a]
Na_2O	-0.28 [b]	n.s	-0.27 [b]	-0.36 [a]	n.s	0.33 [a]	0.24 [b]	n.s
K_2O	n.s	n.s	n.s	-0.37 [a]	n.s	0.24 [b]	n.s	n.s
P	-0.25 [b]	0.29 [a]	n.s	-0.26 [b]	0.26 [b]	n.s	n.s	n.s
Total	1	-0.30 [a]	0.43 [a]	0.38 [a]	-0.38 [a]	-0.34 [a]	n.s	-0.34 [a]

n.s: not significant.

[a] Correlation is significant at 0.05 level.

[b] Correlation is significant at 0.01 level.

Supplementary Table S10 Pearson correlation coefficients between relevant soil properties and Cd and Pb geochemical fractions (% of total) in the soil vertical profiles developed in granite plutons.

Fraction	Targeted forms	Extractants	Equilibration
F1	Soluble fraction	distilled water (pH 7.0)	25ml,shaking for 30mins at room temperature
F2	Ion exchangeable fraction	1M $MgCl_2$•$6H_2O$ (pH 7.0)	25ml,shaking for 30mins at room temperature
F3	Easily mobilizable fraction (sorbed and bound to carbonate)	1M NaAc-HAc (pH 5.0)	25ml,shaking for 60mins at room temperature
F4	Labile organic matter (OM) fraction (sorbed to organic matter)	0.1M $Na_4P_2O_7$•$10H_2O$ (pH 10.0)	50ml,shaking for 40mins at room temperature
F5	Fe-Mn oxides fraction (sorbed and bound to Fe-Mn (hydr)oxides)	0.25M $HONH_3Cl$-HCl	25ml,shaking for 60mins at room temperature
F6	Refractory OM fraction (bound to organic matter, or minor Sulfide forms)	30% H_2O_2 -HNO_3 (pH 2.0)	8ml, placing for 3h at waterbath on 83 ℃ ;shaking 10h at room temperature
F7	Residual fraction (mineral form)	1)HCl-HNO_3-$HClO_4$(1:1:1)<br><br>2)Aqua regia (Conc.HCl+Con.HNO_3)	1) 0.2g residual sample +5ml HCl-HNO_3-$HClO_4$ for Cd and Pb<br>2) 0.2g+20ml Aqua regia for As and Hg

Supplementary Table S11 Sequential metals fraction procedures and targeted metal forms.

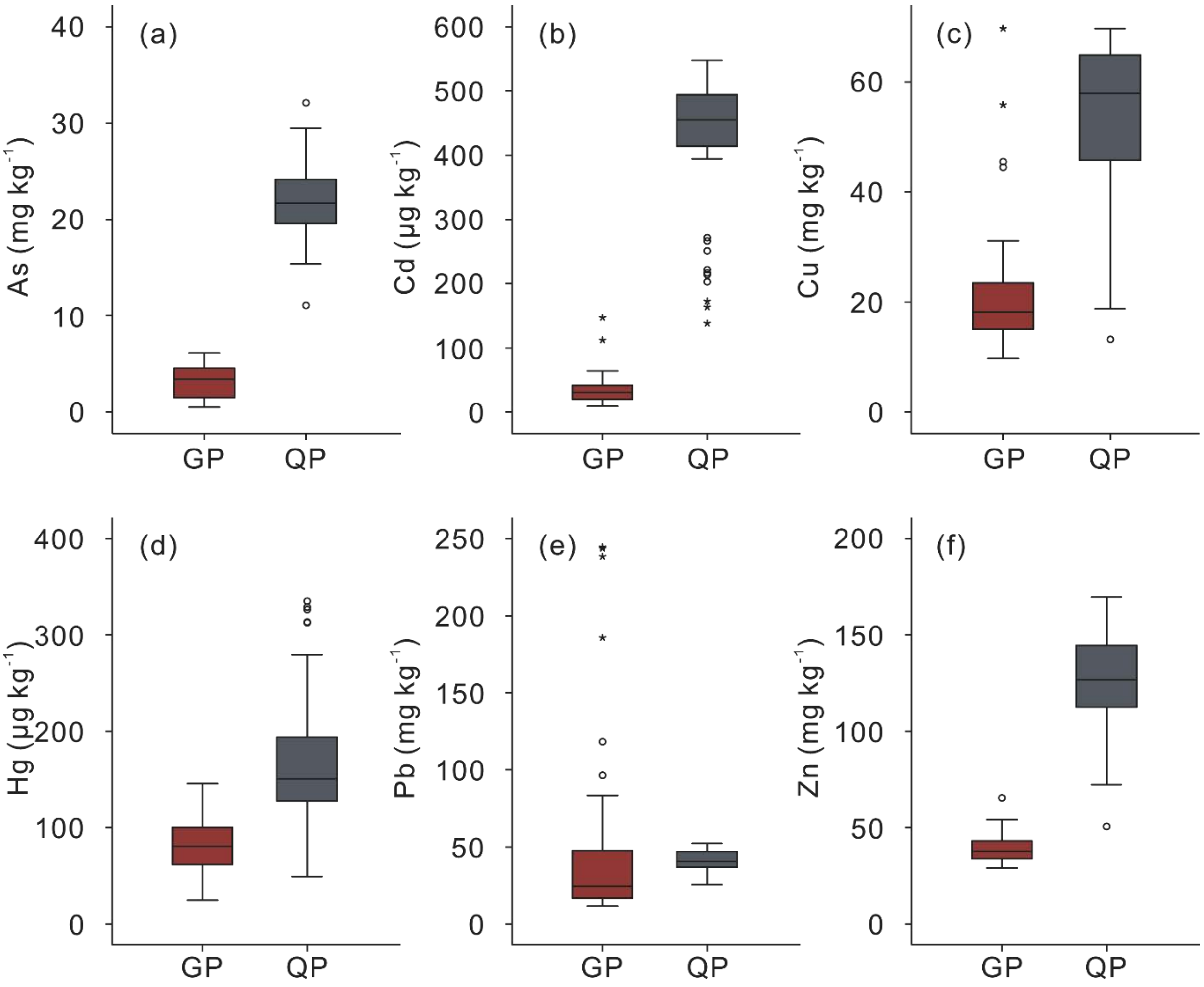

Supplementary Figure S1 The Tukey boxplots of potential toxic elements concentrations in the soil profiles. GP, soil profiles in granite plutons; QP, soil profiles in quaternary sediments.
